# Understanding the Causes of Recent Warming of Mediterranean Waters. How Much Could Be Attributed to Climate Change?

**DOI:** 10.1371/journal.pone.0081591

**Published:** 2013-11-27

**Authors:** Diego Macias, Elisa Garcia-Gorriz, Adolf Stips

**Affiliations:** European Commission, Joint Research Center, Institute for Environment and Sustainability, Water Research Unit, Ispra, Italy; Plymouth University, United Kingdom

## Abstract

During the past two decades, Mediterranean waters have been warming at a rather high rate resulting in scientific and social concern. This warming trend is observed in satellite data, field data and model simulations, and affects both surface and deep waters throughout the Mediterranean basin. However, the warming rate is regionally different and seems to change with time, which has led to the question of what causes underlie the observed trends. Here, we analyze available satellite information on sea surface temperature (SST) from the last 25 years using spectral techniques and find that more than half of the warming tendency during this period is due to a non-linear, wave-like tendency. Using a state of the art hydrodynamic model, we perform a hindcast simulation and obtain the simulated SST evolution of the Mediterranean basin for the last 52 years. These SST results show a clear sinusoidal tendency that follows the Atlantic Multidecadal Oscillation (AMO) during the simulation period. Our results reveal that 58% of recent warming in Mediterranean waters could be attributed to this AMO-like oscillation, being anthropogenic-induced climate change only responsible for 42% of total trend. The observed acceleration of water warming during the 1990s therefore appears to be caused by a superimposition of anthropogenic-induced warming with the positive phase of the AMO, while the recent slowdown of this tendency is likely due to a shift in the AMO phase. It has been proposed that this change in the AMO phase will mask the effect of global warming in the forthcoming decades, and our results indicate that the same could also be applicable to the Mediterranean Sea. Henceforth, natural multidecadal temperature oscillations should be taken into account to avoid underestimation of the anthropogenic-induced warming of the Mediterranean basin in the future.

## Introduction

It has been repeatedly reported in the scientific literature that the water temperature in the Mediterranean Sea has been rising at quite a high rate during the last two decades [1], [2], mirroring the global ocean tendency [3]. This warming trend is observed in remote sensing data [4], field data [5] and model simulations [6], and affects both the surface and deep waters of the entire Mediterranean basin [2].

Changes in sea temperature can modify the sea level through the thermal expansion of seawater [1] and affect deep water formation in this evaporative basin, changing ventilation and renovation times [6] and crucially altering the general current configuration in the region [7]. Marine ecosystems could also suffer changes forced by these physical modifications [8], such as expansion/contraction of habitat distributions, invasion by allochthonous species and changes in primary productivity patterns and fish distributions [9]. Thus, the scientific and social concerns about the possible consequences of the observed changes on the ecosystem functioning and associated services of this heavily populated basin have been quite high [2].

Warming rates of the Mediterranean basin differ geographically [4], [10] and seem to change with time, having been more rapid during the 1990s and slower in the preceding and following decades [5], [6], [11]. Natural temperature oscillations have been proposed as a probable cause of these fluctuations in the Mediterranean water temperature [11], [12], but no quantification of the relative contribution from anthropogenic-induced climate change and from such natural oscillations have been proposed so far.

In the present work, we will combine satellite data and numerical modeling to discern the different forcings contributing to observed and simulated oscillations in Mediterranean Sea Surface Temperature. We will start analyzing available SST data from remote sensors with spectral methods to extract the hidden signals within the total trend. After that, the simulated SST will be validated against satellite data for the observational period (1985 to 2010) in order to determine the performance of the proposed hydrodynamic model. Afterwards, SST predictions from a long-model run covering the last 52 years (1960–2011) will be analyzed using the same spectral techniques and the recovered patterns will be compared with low-frequency, basin-wide oscillations in the North Atlantic region. This shall allow the correct attribution of the experienced warming with respect to climate change. Some statistical projections of SST in the Mediterranean basin for the next future will be presented at the end of the paper.

## Results

**Figure 1:**
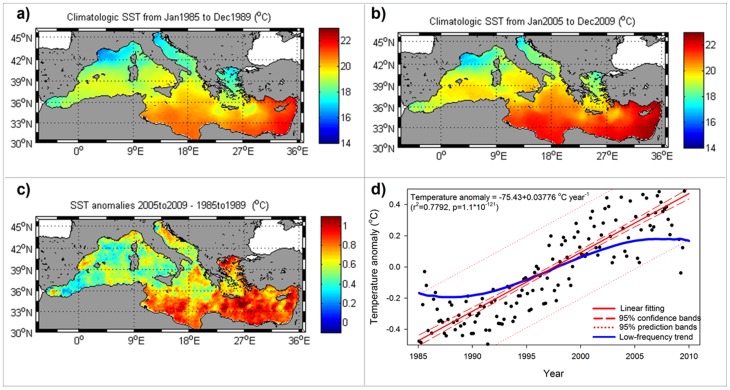
Analysis of satellite sea surface temperature (SST) data in the Mediterranean basin from 1985 to 2009. a) Climatologic SST in the basin between January 1985 and December 1989. b) Climatologic SST in the basin between January 2005 and December 2009. c) Differences in SST between the period from 2000 to 2005 and the period from 1985 to 1989. d) Tendency of the SST time series after removing the annual and semiannual signals by applying the singular spectral analysis (black dots). Linear fitting of the SST tendency (red lines and inserted equation). Main signal within the SST tendency obtained from the singular spectral analysis (blue line).

The general warming trend is easily observed in the available satellite data on sea surface temperature (SST) for the Mediterranean basin. A comparison of the mean SST values in the basin for the first five available years (1985–1989) in the dataset (Fig. 1a) with the mean values for the last five years (2005–2009) (Fig. 1a) reveals generalized warming of the basin (Fig. 1c) during these 25 years, with a mean increase of 0.67°C±0.17°C being detected. As reported previously [4], warming is more acute in the eastern region (latitude >15°E) with a mean increase of 0.77°C±0.16°C and less pronounced in the western basin (mean 0.54°C±0.14°C).

To obtain the SST trend for the 25 years of available satellite data, we applied a singular spectral analysis (SSA) [13] to the monthly mean SST time series to filter the annual and semiannual signals, which are the two dominant types of variability in the series [14]. The observed tendency (black dots in Fig. 1d) represented almost 2% of the total energy in the series and could be fitted to a linear regression (solid red line in Fig. 1d) with a slope of 0.0377°C year^−1^ which corresponds quite well to previously reported values for the mean warming rates in the Mediterranean [4]. However, within the total trend, one mode of variability could be isolated as the third most energetic signal in the series [after annual and semiannual cycles], accounting for 60.3% of the total variability in the trend (blue line in Fig. 1d). This low-frequency signal alone is responsible for a warming of 0.393°C during the analyzed period, which is 58.65% of the total temperature increase. The shape of this curve is sigmoidal, and it shows enhanced warming during the 1990s and a lower slope in the years before and after this period. Henceforth, it appears that this tendency could be part of a long-term oscillation, as proposed previously [5], [12]. Unfortunately, there are no available satellite data of an adequate quality to extend this analysis sufficiently back in time (even if Pathfinder data are available since late 1981).

An alternative to the use of measured data is numerical simulation. We set-up a hydrodynamic model of the entire Mediterranean basin using the General Estuarine Transport Model (GETM) [15] forced by atmospheric inputs (wind, solar radiation, heat fluxes and precipitation) provided by the European Centre for Medium Weather Forecast (ECMWF) covering the period from 1960 to 2011 (see details in Material and Methods section). Mean monthly SST values for the entire Mediterranean basin were computed from the 3D fields provided by the model.

**Figure 2:**
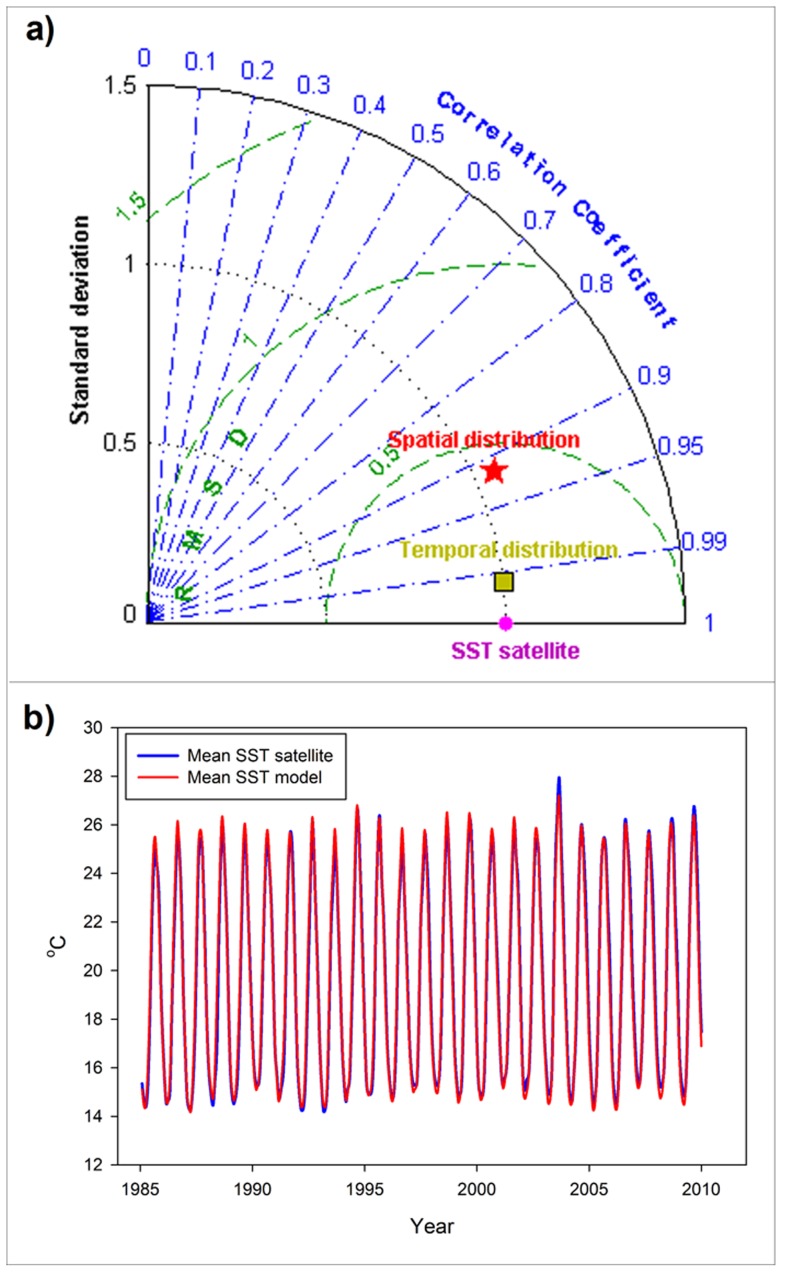
Satellite data – model simulation comparison for the period from 1985 to 2009. a) Taylor Diagram of the model-satellite data comparison. The red star corresponds to the spatial distribution comparison. The yellow square corresponds to the temporal evolution comparison. b) Time series of the observed (from satellite) monthly mean SST (blue line) and simulated monthly mean SST (red line) during the period from 1985 to 2009.

The results of the model corresponding to the years between 1985 and 2009 were used to validate our model against satellite values (Fig. 2). The climatological SST distribution during this time period was reasonably simulated by the model, showing a pearson-correlation coefficient with the observed patterns of r = 0.927 (p<0.001), a mean difference of 0.18°C and a very similar standard deviation (representing spatial heterogeneity) in the model and data (Fig. 2a). For the purpose of the analysis presented here, however, we are more interested in how the model reproduces the temporal evolution of the mean SST observed in the satellite data (Fig. 2b). In this case, mean SST values are very similar, with a mean difference between the model and data of only 0.0096°C being detected throughout the analyzed time-span. The pearson-correlation coefficient is r = 0.9935 (p<0.001), with the standard deviation (now representing temporal variability) being almost identical in the model and data (Fig. 2A).

**Figure 3:**
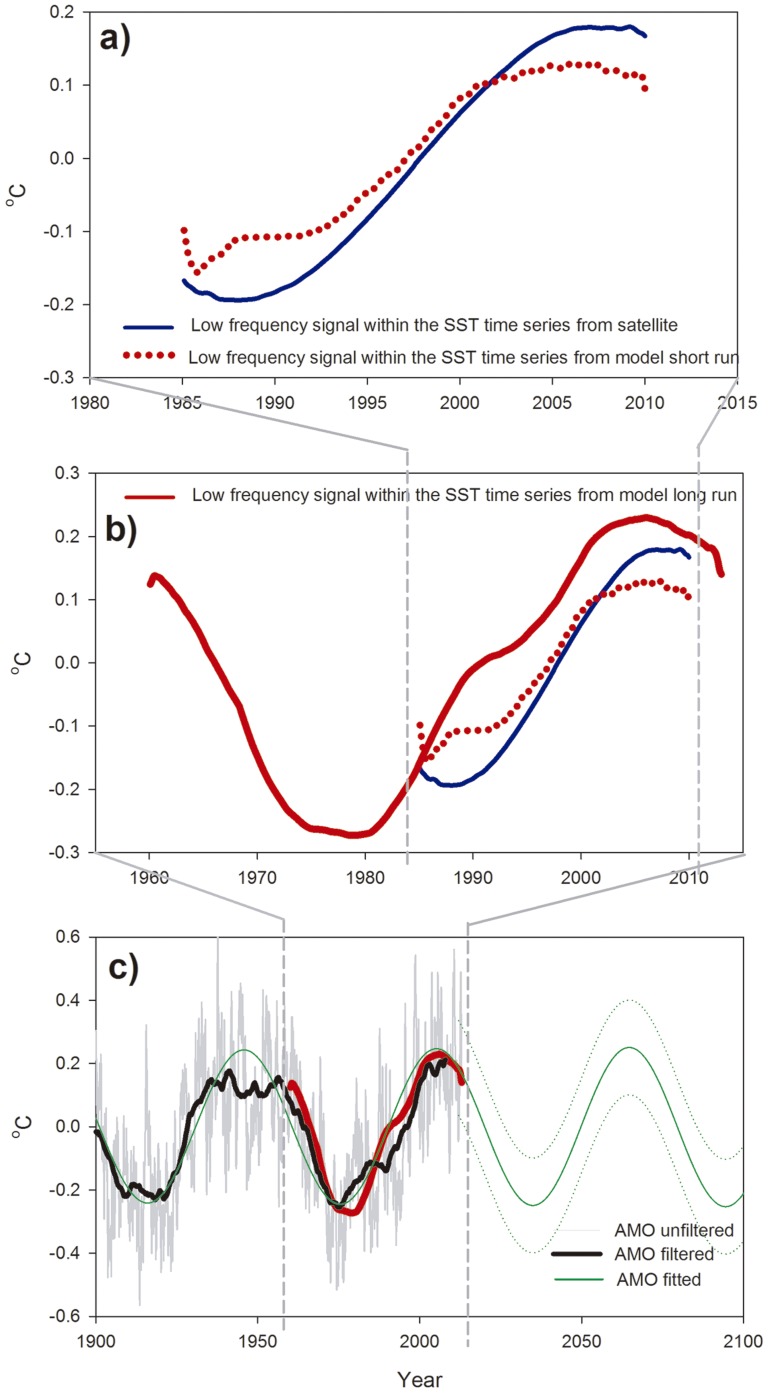
Spectral analysis of the model simulations. a) Low-frequency signal obtained from the monthly simulated SST from 1985–2009 (red dotted line). Low-frequency signal obtained from the monthly satellite data (blue line, the same as shown in Fig. 1b). b) Low-frequency signal obtained from the monthly simulated SST from 1960–2011 (red solid line). For comparison, signals from Fig. 3a have been also included. c) Unfiltered (thin grey line) and filtered (bold black line) Atlantic Multidecadal Oscillation index plotted together with the low-frequency signal obtained from the long model run. The AMO fitted to a sinusoidal equation (see Material and Methods) is shown from 1900 to 2100 (solid green line) along with the 95% confidence prediction bands (dotted green lines).

When the same SSA technique is applied to the modeled mean SST time series (red line in Fig. 2b) a low-frequency signal is again found as the third most energetic signal in the series (red dotted line in Fig. 3a). The shape and amplitude of this signal is very similar to that obtained from the satellite data (also included in Fig. 3a for comparison, blue line) and indicates that the model is a good descriptor of the real-time variability of the SST in the Mediterranean basin. This result provides some confidence in the model's performance and, thus, allows us to apply the same SSA to the whole time series of mean SST obtained for the 52 years of the simulation (red continuous line in Fig. 3b). With this longer time series, the extracted signal, which is, again, the third most energetic one, clearly resembles a low-frequency oscillation with a period of 50–60 years, showing minimum values in the mid-1970s, a sustained increase starting at approximately 1980 and a later decline at the end of the time series (from 2005 onwards). The shape of this curve is in very good agreement with previous reports on the evolution of SST anomalies in the Mediterranean basin [11], [12].

If this signal is superimposed on the Atlantic Multidecadal Oscillation (AMO) index (gray and black lines in Fig. 3c), which constitutes one of the most important natural modes of climate variability in the North Atlantic, a clear concordance is found [16]. The temporal evolution of the AMO and the low-frequency SST signal during the last 52 years is remarkably similar (with a pearson-correlation coefficient of r = 0.9547, p<0.001), as is the range of variation in both variables (Fig. 3c).

## Discussion

The AMO is a natural oscillation of the SST on the North Atlantic region [16] that is consistently found in instrumental records during the last 150 years [17] and is detected in paleorecords for the last 8,000 years [18]. This long-term oscillation has been shown to have decisively influenced temperatures in the North Atlantic during the last several decades [19] and to interact with the global climate [20], [21]. The same situation appears to hold for the Mediterranean Sea, as, throughout the available time span of satellite data, the low-frequency signal identified with the AMO is responsible for more than half (58.65%) of the total warming observed (Fig. 1d). Thus, only 41.35% of total temperature increase in the basin during the last 25 years could be attributed to anthropogenic-induced climate warming.

A low-frequency oscillation resembling the AMO variability has, indeed, been recently described in reconstructed time-series of Mediterranean SST [12]. However, this is the first time that a quantification of the importance of such oscillation in the warming rate of the basin has been performed. Moreover [12] discussed about the causes of such coherent temperature oscillations in the North Atlantic and the Mediterranean Sea. They proposed two mechanisms for such a multidecadal forcing, (*i*) the contribution of the Mediterranean Outflowing Water (MOW) to the thermohaline circulation of the North Atlantic and (*ii*) an atmospheric forcing that creates simultaneous temperatures oscillations in both basins. As we are not considering any variability in the Atlantic boundary of our model (see description of boundary conditions in Material and Methods) our results suggest that the atmospheric origin is, indeed, the most probable cause of the coherent pattern in both basins.

**Figure 4:**
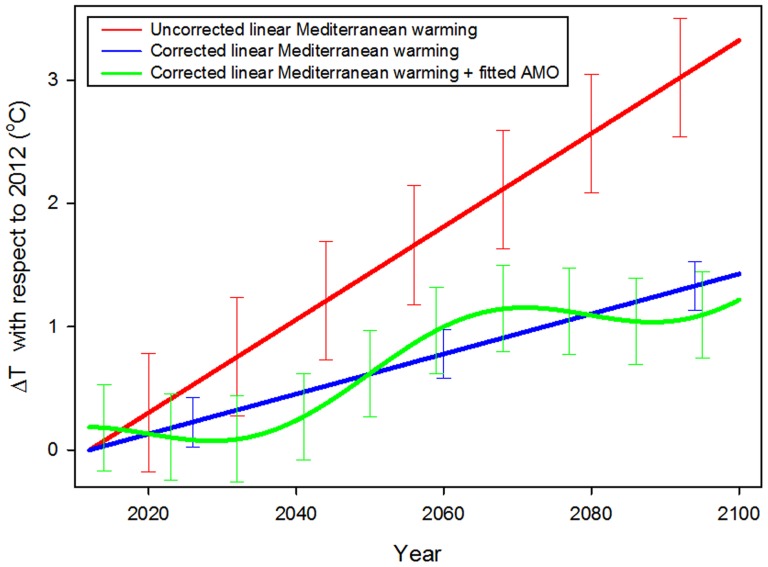
Statistical projections of SST in the Mediterranean basin from the present to 2100. Red line, linear trend estimate derived from the last 25 years of satellite observations (see Fig. 1d). Blue line, SST linear trend equal to the residual tendency in the satellite data during the last 25 years after removing the low-frequency signal revealed by SSA. Green line, SST trend obtained by adding the fitted AMO (green line in Fig. 3c) to the corrected linear trend. Error bars computed from the 95% confidence prediction bands of the fittings (see Figs 1 and 3) are also shown.

The strong influence of the AMO on the Mediterranean SST tendency must, then, be considered when evaluating projections of temperature evolution for the forthcoming decades. The AMO should shift towards a cold phase in the coming years [19], as observed in the fitted curve (green line) presented in Fig. 3c. If the recent total linear trend observed in the satellite data (red solid line in Fig. 1d) is used to estimate SST anomalies in the Mediterranean for the next century, a total increase of nearly 3.4°C can be computed for year 2100 (red line, Fig. 4). If, instead, only 41.35% of the total trend is used as an estimate of anthropogenic-induced warming the expected SST increase is approximately 1.4°C for 2100 (blue line, Fig. 4). However, when the corrected warming trend is added to the AMO projection (green line, Fig. 4), periods of nearly no increase (corresponding to low phases of the AMO) alternate with periods of enhanced warming during the next 100 years.

This statistical projection indicates that, because of the interaction between global warming and natural variability (green line in Fig. 4), during the next decades (at least until 2030) the SST in the Mediterranean could remain quite constant. In fact, the low-frequency signal obtained from the long model run (Fig. 3b) even shows a declining trend in its last section, starting from 2005. This cooling tendency has been observed previously for the same period in field data from the western Mediterranean Sea [5] and demonstrates the importance of considering the variability in the AMO to avoid underestimating the importance of the anthropogenic-induced warming in the Mediterranean basin.

## Materials and Methods

### Satellite data

In the present study, we employed the 4 km Advanced Very High-Resolution Radiometer (AVHRR) Pathfinder Version 5 sea surface temperature (SST) dataset. AVHRR Oceans Pathfinder SST data were obtained from the Physical Oceanography Distributed Active Archive Center (PO.DAAC) at the NASA Jet Propulsion Laboratory, Pasadena, CA (http://podaac.jpl.nasa.gov, accessed 2011 Jun 25). This dataset represents a reanalysis of historical AVHRR data that have been improved through extensive calibration and validation and using any other available information to yield a consistent research-quality time series for global climate studies. This SST time series represents the longest continuous set of physical measurements for the global ocean obtained from space.

### Atlantic Multidecadal Oscillation (AMO) index

The AMO is defined as the sea surface temperature (SST) anomaly from 0–70°N over the North Atlantic region [20], linearly detrended to account for the increase in temperature associated with anthropogenic climate change. Monthly values of the index were obtained from the National Oceanic and Atmospheric Administration (NOAA) web server at http://www.esrl.noaa.gov/psd/data/timeseries/AMO/(accessed 2013 July 8). These values are computed by NOAA from the Kaplan SST dataset, which consists of gridded global SST anomalies from 1856 to the present day derived from the UK Met Office SST data, to which sophisticated statistical techniques have been applied to it to fill in gaps [22].

Smoothed monthly AMO values from 1900 to 2010 (bold black line, Fig. 3c) were fitted to a simple sinusoidal curve (green line, Fig. 3c) according to the following equation:
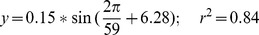



This fitting allows the projection of an AMO-like oscillation into the next century to obtain the estimated evolution of the SST anomaly for the future (green line, Fig. 4).

### Singular Spectral Analysis (SSA)

SSA is designed to extract information from short and noisy time series and, thus, provide insight into the unknown, or only partially known, dynamics of the underlying system that generated the series [23]. This methodology is analogous to applying an extended empirical orthogonal function (EEOF) analysis to successive lags of a univariate series and is equivalent to representing the behavior of the system by a succession of overlapping “views” of the series through a sliding *n*-point window [24]. In so doing, the SSA allows the decomposition of the time series into a sequence of elementary patterns of behavior that are classified as either trends or oscillatory patterns.

From this decomposition into eigenvalues, it is possible to reconstruct each of the individual signals by adding the corresponding eigenvectors to the sample mean [24]. This SSA method has proved to be a powerful tool for analyzing time-series of oceanographic variables such as SST [12], chlorophyll-a [13] or the upwelling intensity [14].

For the analysis presented here, the annual and semiannual signals [accounting for almost 98% of total variability] were removed from the time series to obtain the underlying trend (see dots in Fig. 1d). Within this trend (representing approximately 2% of the total variability), the most energetic eigenvalue (accounting for 58% of the energy of the trend) corresponds to the low-frequency signal identified with a blue line in Fig. 1D.

### Hydrodynamic model

We use the 3-D General Estuarine Transport Model (GETM) to simulate the hydrodynamics in the Mediterranean Sea. GETM solves the three-dimensional hydrostatic equations of motion applying the Boussinesq approximation and the eddy viscosity assumption [25]. For a detailed description of the GETM equations, see [15] and http://www.getm.eu. The configuration of the Mediterranean Sea has a horizontal resolution of 5′×5′ and includes 25 vertical layers, within a time window from January 1960 to December 2011. ETOPO1 is used to build the bathymetric grid by averaging to the corresponding horizontal resolution of the model grid. The salinity and temperature climatologies required at the start of model integration were obtained from the Mediterranean Data Archaeology and Rescue-MEDAR/MEDATLAS database (http://www.ifremer.fr/medar/, accessed 2012 May 18). The Strait of Gibraltar is prescribed as an open boundary and the Dardanelles inflow is treated as a riverine inflow within the basin. The current configuration of the model includes 37 rivers discharging along the Mediterranean coast. The corresponding river discharges are derived from the Global River Data Center (GRDC, Germany) database. The GETM runs for the Mediterranean Sea are forced at surface every 6 hours with ECMWF reanalysis products. Specifically, we use the ECMWF ERA40 reanalysis products from 1960 to 1978 and the ERA-Interim products from 1979 to 2011. The ECMWF ERA Reanalysis data are available from the ECMWF data server (http://www.ecmwf.int, accessed 2012 Jun 25).

### Model-satellite comparison

Satellite SST data were interpolated to the model grid to obtain consistent data matrices. Water temperatures were extracted from the 3D model fields at each grid point at a depth of 4 meters, as it has been demonstrated that the best fitting between Pathfinder and field data occurs at this depth [26]. Day and night measurements from Pathfinder were used to calculate mean satellite temperature as model means were computed using the entire daily cycle.
